# A data-driven study of Chinese participants' social judgments of Chinese faces

**DOI:** 10.1371/journal.pone.0210315

**Published:** 2019-01-04

**Authors:** Hongyi Wang, Chengyang Han, Amanda C. Hahn, Vanessa Fasolt, Danielle K. Morrison, Iris J. Holzleitner, Lisa M. DeBruine, Benedict C. Jones

**Affiliations:** 1 School of Psychology and Cognitive Science, East China Normal University, Shanghai, China; 2 College of Education, Zhejiang University, Hangzhou, China; 3 Department of Psychology, Humboldt State University, Arcata, CA, United States of America; 4 Institute of Neuroscience and Psychology, University of Glasgow, Glasgow, United Kingdom; University of Wroclaw, POLAND

## Abstract

Social judgments of faces made by Western participants are thought to be underpinned by two dimensions: valence and dominance. Because some research suggests that Western and Eastern participants process faces differently, the two-dimensional model of face evaluation may not necessarily apply to judgments of faces by Eastern participants. Here we used a data-driven approach to investigate the components underlying social judgments of Chinese faces by Chinese participants. Analyses showed that social judgments of Chinese faces by Chinese participants are partly underpinned by a general approachability dimension similar to the valence dimension previously found to underpin Western participants’ evaluations of White faces. However, we found that a general capability dimension, rather than a dominance dimension, contributed to Chinese participants’ evaluations of Chinese faces. Thus, our findings present evidence for both cultural similarities and cultural differences in social evaluations of faces. Importantly, the dimension that explained most of the variance in Chinese participants’ social judgments of faces was strikingly similar to the valence dimension previously reported for Western participants.

## Introduction

People automatically and rapidly evaluate faces on a variety of traits (e.g., trustworthiness, attractiveness [1–3]). These stereotypic evaluations can have substantial effects on people’s behavior (e.g. [4]). Thus, understanding factors that influence our social evaluations of faces can provide important insights into one route through which social stereotypes can influence social interaction [5–7].

Principal Component Analysis (PCA) of multiple trait ratings has revealed that social judgments of faces can be reduced to two main orthogonal dimensions: valence and dominance ([5–7], but see [8,9] for evidence of an additional youthful-attractiveness dimension under some circumstances). The valence dimension is positively correlated with all positive traits (e.g., perceived attractiveness and trustworthiness) and negatively correlated with all negative traits (e.g., perceived weirdness and meanness) and can be interpreted as valence evaluations [5–7]. Because the valence dimension is highly correlated with traits such as perceived trustworthiness, it is thought to reflect perceptions of an individual’s general approachability [5–7]. The second dimension is highly correlated with traits such as perceived dominance and perceived aggressiveness and thus is labelled the dominance dimension. The dominance dimension is thought to reflect perceptions of an individual’s capacity to cause physical harm [5–7]. These two components can together reproduce important social judgments, such as threat, as the valence dimension functions to signal the target’s intention and the dominance dimension functions to signal the target’s physical strength to cause the harm.

The studies described above were all conducted in Western cultures. However, some previous research suggests that Western and Eastern participants process faces differently. For example, Jack et al. found that Eastern and Western participants represented facial expressions of emotions in different ways [10]. More recently, Han et al. found that Eastern and Western participants’ showed different preferences for color information when judging the attractiveness and health of faces [11]. These findings suggest that the two-dimensional model of face evaluation previously reported for Western participants may not necessarily generalize to judgments of faces by Eastern participants.

Only one study has directly addressed this issue. Sutherland et al. used a data-driven approach to investigate the perceptual dimensions that underpinned Chinese participants’ social judgments of highly variable (unstandardized or ‘ambient’) images of Chinese faces [9]. They found that these social judgments were underpinned by three dimensions representing general approachability, youthful-attractiveness, and capability, respectively. Importantly, the general approachability dimension explained most of the variance in evaluations and was strikingly similar to the valence dimension reported in previous studies of Western participants’ evaluations of White faces [5–9]. Thus, Sutherland et al. concluded that there were broad similarities in the perceptual dimensions underpinning both Western participants’ evaluations of White faces and Chinese participants’ evaluations of Chinese faces [9]. However, Sutherland et al. [9] did not investigate any dominance-related traits in their study, since these traits were not revealed by their bottom-up approach. This potentially raises questions about the absence of the evidence for a dominance dimension in their study, since Oosterhof and Todorov [6] included dominance as a trait in their analyses because of its theoretical importance (see, e.g., [12]), despite it also not emerging from their free descriptions.

The current study independently used a data-driven approach similar to that used by Sutherland et al. [9] to investigate the components underlying social judgments of Chinese faces by Chinese participants. By contrast with Sutherland et al. [9], who used highly variable, unstandardized images of faces, we used standardized face stimuli more similar to those used by Oosterhof and Todorov [6] allowing for a more direct comparison between their two-dimensional model and our results. By contrast with Sutherland et al. [9], faces were rated for dominance, in addition to the traits revealed in free descriptions, which could address the issue whether the dominance dimension contributes to Chinese participants’ evaluation of Chinese faces.

## Methods

### Stimuli

To collect face stimuli for the project, we recruited 50 Chinese men (mean age = 24.39 years, SD = 3.52 years) and 50 Chinese women (mean age = 23.94 years, SD = 2.63 years). These men and women were all born in China, but currently resided in the UK (mean number of years in the UK = 1.05 years, SD = 0.93 years). Participants provided informed written consent before participating. University of Glasgow’s Science and Engineering Ethics Committee and East China Normal University’s Committee on Human Research Protection approved all aspects of the study. Participants whose photographs are shown in this paper provided written consent for their photographs to be used in original or altered forms for illustrative purposes.

These men and women first cleaned their face with hypoallergenic face wipes to remove any make-up. Photographs were taken a minimum of 10 minutes later in a small windowless room against a constant background, and under standardized diffuse lighting conditions. The men and women were instructed to pose with a neutral expression. Camera-to-head distance and camera settings were held constant. Men and women wore a white smock covering their clothing when photographed to control for possible effects of reflectance from clothing. Six photographs of each individual were taken simultaneously from a variety of angles. Only the front view pictures were used in the study. Each image was standardized on pupil positions and masked so that hairstyle and clothing were not visible.

### Free descriptions of faces

To identify traits that are automatically inferred from neutral faces, another 10 Chinese men (mean age = 24.12 years, SD = 2.22 years) and 22 Chinese women (mean age = 24.05 years, SD = 4.61 years) were recruited to give unconstrained descriptions of faces (mean number of years in the UK = 0.91 years, SD = 1.30 years). The participants were all native Chinese speakers and the instructions of the task were written in Chinese. Following Oosterhof and Todorov [6], participants were instructed to describe 50 male and 50 female Chinese faces using their own words in Chinese (1 to 5 adjectives for each face). Adjectives used 5 times or more were then entered into hierarchical cluster analysis (~72% of the adjectives were entered into the analyses) (e.g.,[13]). The semantic distances among words were determined by word vectors pre-trained on Chinese Wikipedia using fastText [14]. These analyses produced 14 clusters. We named each cluster using the adjective that had been used most frequently in that cluster. These 14 traits are 好看(attractiveness), 开朗(cheerfulness), 普通(commonness), 呆(dullness), 平易近人(easygoingness), 友善(friendliness), 单纯(ingenuousness), 聪明(intelligence), 善良(kindness), 忧郁(melancholy), 乐观(optimism), 严肃(seriousness), 可信(trustworthiness), 凶(viciousness).

### Face ratings

Another 10 Chinese men (mean age = 22.20 years, SD = 4.05 years) and 10 Chinese women (mean age = 19.30 years, SD = 1.34 years), all of whom were born and resided in China, rated the face stimuli on all 14 traits using 1 (low) to 7 (high) rating scales. Each participant rated male and female stimuli on all 14 traits. Different traits were rated in separate blocks of trials and male and female stimuli were rated in separate blocks of trials [7,15]. Both block order and trial order within each block were fully randomized. Following other studies that investigated the components underlying social judgments, traits were not defined for participants [6, 7,15].

## Results

Analyses were conducted using R [16] with the package Psych [17]. Inter-rater agreement, estimated by Cronbach’s alpha, was high for all trait ratings (i.e., Cronbach’s alpha >.70), with the exception of commonness and dullness ratings. These low-reliability traits were excluded from our main analyses (following [6,7]). Descriptive statistics for each trait are shown in Table 1.

**Table 1:** Descriptive statistics for all traits.

	Male faces			Female faces		
**Trait**	***α***	***M***	***SD***	***α***	***M***	***SD***
**Attractiveness**	0.84	2.97	0.53	0.88	3.33	0.59
**Cheerfulness**	0.85	3.75	0.59	0.89	3.86	0.68
**Commonness**	0.43	4.66	0.36	0.19	4.68	0.31
**Dullness**	0.61	4.15	0.48	0.65	3.90	0.48
**Easygoingness**	0.82	3.80	0.56	0.89	4.11	0.66
**Friendliness**	0.85	3.74	0.62	0.85	4.15	0.61
**Ingenuousness**	0.78	3.83	0.57	0.78	4.16	0.51
**Intelligence**	0.83	3.84	0.57	0.76	3.94	0.49
**Kindness**	0.82	3.87	0.60	0.77	4.24	0.46
**Melancholy**	0.82	3.83	0.55	0.84	3.83	0.65
**Optimism**	0.86	3.92	0.60	0.88	4.07	0.66
**Seriousness**	0.84	4.32	0.64	0.89	4.06	0.71
**Trustworthiness**	0.75	3.83	0.53	0.71	4.18	0.41
**Vicious**	0.88	3.75	0.69	0.86	3.71	0.69

First, we calculated the mean rating for each face separately for each trait. We then analyzed these mean ratings using principal component analysis (PCA) with no rotation to reveal the components underlying ratings of social stimuli (following [5,6,7,15]). Components with eigenvalues greater than 1 were extracted and ratings of male and female faces were analyzed separately.

**Fig 1:**
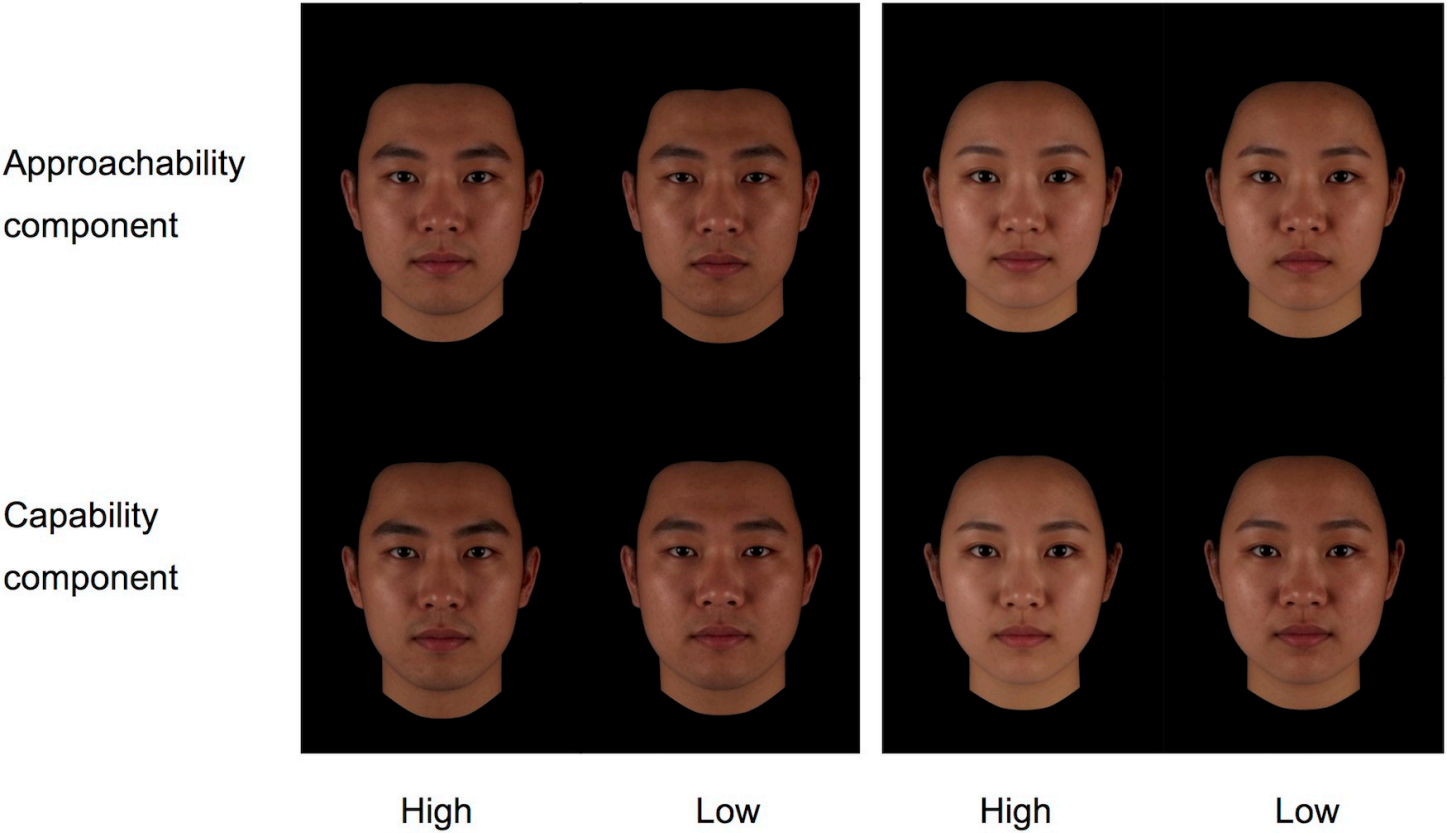
Composites of 15 faces that scored highest and lowest in the two components of male and female faces.

Both analysis of ratings of male and female faces extracted two components with eigenvalues greater than 1. For male faces, the first component explained approximately 68% of the variance in ratings and was highly correlated with friendliness, easygoingness, optimism and kindness. Similarly, for female faces, the first component explained approximately 73% of the variance in ratings and was also highly correlated with easygoingness, friendliness, optimism, and kindness. The second component explained approximately 12% of the variance in ratings of male faces and was highly correlated with intelligence and attractiveness. Similarly, the second component explained approximately 12% of the variance in ratings of female faces and was also highly correlated with intelligence and attractiveness. The component matrix is shown in Table 2 for both male and female faces. And Fig 1 shows the average composites of the 15 faces scored high and low in the two components for both male and female faces.

**Table 2:** Component matrix for principal component analysis of male and female face ratings.

Trait	Male face		Female face	
	*Approachability* *component*	*Capability* *component*	*Approachability* *component*	*Capability* *component*
**Attractiveness**	0.631	0.689	0.438	0.824
**Cheerfulness**	0.894	0.068	0.929	-0.087
**Easygoingness**	0.930	-0.100	0.957	0.023
**Friendliness**	0.959	-0.080	0.940	-0.104
**Ingenuousness**	0.874	-0.212	0.813	-0.168
**Intelligence**	0.397	0.847	0.436	0.828
**Kindness**	0.907	-0.062	0.920	-0.054
**Melancholy**	-0.826	0.010	-0.893	0.037
**Optimism**	0.929	0.072	0.928	-0.043
**Seriousness**	-0.756	0.380	-0.903	0.186
**Trustworthiness**	0.783	0.027	0.870	-0.020
**Vicious**	-0.850	0.250	-0.919	0.131

Although our initial study of free descriptions of faces did not produce a cluster labelled dominance, we had the faces rated for dominance along with the other 14 traits that emerged from the free descriptions. We did this because Oosterhof and Todorov [6] had included dominance as a trait in their analyses because of its theoretical importance in the social perception literature (see, e.g., [12]), although it also did not emerge from their free descriptions. Repeating PCAs of male and female faces with dominance included produced a similar pattern of results (i.e., both analyses produced *approachability* and *capability* components similar to those reported above). The *approachability* component explained approximately 67% of the variance in ratings for male faces and 72% of the variance in ratings for female faces respectively. The *capability* component explained approximately 12% of the variance in ratings for male faces and 11% of the ratings for female faces respectively. The component matrix is shown in Table 3 for both male and female faces.

**Table 3:** Component matrix for principal component analysis of male and female face ratings including dominance ratings.

Trait	Male face		Female face	
	*Approachability* *component*	*Capability* *component*	*Approachability* *component*	*Capability* *component*
**Attractiveness**	0.612	0.701	0.425	0.826
**Cheerfulness**	0.886	0.112	0.924	-0.059
**Dominance**	-0.761	0.336	-0.903	0.163
**Easygoingness**	0.939	-0.089	0.955	0.042
**Friendliness**	0.956	-0.035	0.946	-0.092
**Ingenuousness**	0.876	-0.167	0.818	-0.159
**Intelligence**	0.380	0.824	0.420	0.839
**Kindness**	0.904	-0.017	0.921	-0.039
**Melancholy**	-0.817	-0.042	-0.884	0.005
**Optimism**	0.919	0.112	0.925	-0.017
**Seriousness**	-0.773	0.376	-0.901	0.160
**Trustworthiness**	0.773	0.082	0.875	-0.013
**Vicious**	-0.869	0.259	-0.925	0.121

Principal Component Analyses carried out separately for ratings from male and female participants for male and female faces respectively did not alter the pattern of results (i.e., all analyses produced similar *approachability* and *capability* components to those reported above). The results of the additional analyses are reported in S1 File.

## Discussion

The current study used a data-driven approach to investigate the dimensions underlying social judgments of Chinese faces by Chinese participants. Principal component analysis of trait ratings of both male and female faces produced a two-component model that explained 80% of the variance in ratings of male faces and 85% of the variance in ratings of female faces. Replicating Sutherland et al.’s recent study of Chinese participants’ ratings of Chinese faces [9], the first component was highly correlated with traits such as easygoingness and friendliness and appeared to reflect a general approachability dimension similar to that reported previously for Western participants’ ratings of White faces [5–7]. Thus, our results present further evidence that Chinese participants, like Western participants, primarily assess faces on general approachability / valence. As the approachability / valence dimension is thought to reflect the target’s intentions (harmful vs harmless) [5–8], the cross-cultural agreement on this dimension suggests that judging strangers on this dimension may have adaptive significance for both Chinese and UK participants.

Previous research into White participants’ judgments of White faces revealed the existence of a prominent dominance dimension on which individuals are evaluated for their perceived capacity to inflict physical harm. Like Sutherland et al. [9], we saw no evidence that Chinese participants assessed faces on this dimension. Instead, we observed a second component that was highly correlated with traits such as perceived intelligence and attractiveness. This dimension appears to be similar to the *capability* component reported by Sutherland et al. [9]. Thus, our results support Sutherland et al.’s conclusion that some differences exist in the dimensions along which Chinese and Western participants evaluate faces. While the dominance dimension may function to signal physical strength, the capacity dimension may reflect another kind of strength, intellectual strength, which is important for individuals to survive and obtain resources and high social status. Evaluations of faces on the capacity dimension might function to identify valuable partners or leaders, which might be of greater importance for collective societies, such as in China [18], than for more individualistic societies.

Using unstandardized images with a diverse age range, Sutherland et al. found that both Chinese and Western participants assessed faces partly on a youthfulness-attractiveness dimension [8,9]. We saw no evidence for such a dimension in the current study, suggesting it may be somewhat specific to rating contexts where a wide age range of individuals is shown. Indeed, they included age as a trait in their analyses, which loaded strongly on the youthfulness-attractiveness dimension [8,9]. Our results do suggest, however, that the capability dimension reported by Sutherland et al. [9] and not evident in previous work using Western participants to assess White faces [6] is not due to differences in the type and age range of the stimuli used and is likely to be a robust feature of Chinese participants’ evaluations of Chinese faces.

There are limitations to the current study that should be acknowledged. First, we had each participant rate faces on all traits, rather than on only one single trait. Although this procedure can reduce the variance at the rater level [19], it might cause other potential issues, such as the low reliability of ratings possibly resulting from participant fatigue. Second, that the initial free descriptions of faces were collected from Chinese participants who were residing in the UK (years of stay in the UK: mean = 0.91, SD = 1.30, minimum = 0.02, maximum = 5.64, median = 0.55), rather than in China might influence their descriptions of the faces. Third, although the current study provides a direct comparison with well-established, two-dimensional model of face evaluation previously reported for Western participants [5–7], our study does not provide a cross-cultural comparison. In addition, consistent with previous studies into the underlying structure of social judgments of faces in general [6,9], we did not distinguish between either sexes of stimuli or sexes of raters when analyzing the free descriptions of faces. However, it is possible that the sex of raters or the sex of face stimuli will influence how people describe faces. It would be useful for future work to address these issues.

In summary, our analyses present further evidence that social judgments of Chinese faces by Chinese participants are underpinned by a general approachability dimension similar to the valence dimension previously found to underpin Western participants’ evaluations of White faces. By contrast with previous results for Western participants’ evaluations of White faces, we found no evidence that dominance perceptions play an important role in Chinese participants’ evaluations of Chinese faces, instead finding evidence for a role for a general capability dimension. Thus, like Sutherland et al.’s [9] recent results, our findings present evidence for both cultural similarities and cultural differences in social evaluations of faces.

## Supporting information

S1 File Additional analyses for face ratings from male and female participants. (HTML)
