# Supplementary material for: A data-driven study of Chinese participants' social judgments of Chinese faces
Source: PLoS One. 2019 Jan 4;14(1):e0210315. doi: 10.1371/journal.pone.0210315 (PMC6319767; doi:10.1371/journal.pone.0210315)
Supplement: S1 File — (HTML) [file pone.0210315.s001.html]

Additional analyses for face ratings from male and female participants


Code 

- Show All Code
- Hide All Code
- Download Rmd

# Additional analyses for face ratings from male and female participants

#### *Hongyi Wang et al.*

- Rating task demographics
- Cronbach’s alpha of ratings
- Descriptive statistics for all traits
- Principal Components Analyses
  - PCA male faces, including dominance
  - PCA female faces, including dominance
  - PCA male faces, not including dominance
  - PCA female faces, not including dominance
- Additional analyses for face ratings from male and female participants
  - PCA male faces by male raters, including dominance
  - PCA male faces by female raters, including dominance
  - PCA female faces by male raters, including dominance
  - PCA female faces by female raters, including dominance
  - PCA male faces by male raters, not including dominance
  - PCA male faces by female raters, not including dominance
  - PCA female faces by male raters, not including dominance
  - PCA female faces by female raters, not including dominance


```
library(psych)
library(broman) # for sensible rounding function
library(knitr)
library(tidyverse)
```


```
data_rating <- read_csv("chinese_rating_raw.csv")
```


### Rating task demographics


```
### get age and sex info 
data_rating %>% 
  group_by(participant_id, part_sex) %>%
  summarise(age=mean(part_age)) %>%
  ungroup() %>%
  group_by(part_sex)%>%
  summarize(age.m=myround(mean(age),2),
            age.sd=myround(sd(age),2),
            count=n()) %>%
  ungroup() %>%
  kable()
```


```
|part_sex |age.m |age.sd | count|
|:--------|:-----|:------|-----:|
|female   |19.30 |1.34   |    10|
|male     |22.20 |4.05   |    10|
```

## Cronbach’s alpha of ratings


```
|Trait           |Male Face |Female Face |
|:---------------|:---------|:-----------|
|Attractiveness  |0.84      |0.88        |
|Cheerfulness    |0.85      |0.89        |
|Commonness      |0.43      |0.19        |
|Dominance       |0.81      |0.87        |
|Dullness        |0.61      |0.65        |
|Easygoingness   |0.82      |0.89        |
|Friendliness    |0.85      |0.85        |
|Ingenuousness   |0.78      |0.78        |
|Intelligence    |0.83      |0.76        |
|Kindness        |0.82      |0.77        |
|Melancholy      |0.82      |0.84        |
|Optimism        |0.86      |0.88        |
|Seriousness     |0.84      |0.89        |
|Trustworthiness |0.75      |0.71        |
|Vicious         |0.88      |0.86        |
```

## Descriptive statistics for all traits


```
|trait_rating    |female.mean |female.sd |male.mean |male.sd |t.value |p.value |
|:---------------|:-----------|:---------|:---------|:-------|:-------|:-------|
|Attractiveness  |3.33        |0.59      |2.97      |0.53    |3.17    |0.002   |
|Cheerfulness    |3.86        |0.68      |3.75      |0.59    |0.84    |0.400   |
|Commonness      |4.68        |0.31      |4.66      |0.36    |0.24    |0.812   |
|Dominance       |3.57        |0.69      |4.07      |0.63    |-3.74   |0.000   |
|Dullness        |3.90        |0.48      |4.15      |0.48    |-2.59   |0.011   |
|Easygoingness   |4.11        |0.66      |3.80      |0.56    |2.54    |0.013   |
|Friendliness    |4.15        |0.61      |3.74      |0.62    |3.27    |0.001   |
|Ingenuousness   |4.16        |0.51      |3.83      |0.57    |2.98    |0.004   |
|Intelligence    |3.94        |0.49      |3.84      |0.57    |0.93    |0.354   |
|Kindness        |4.24        |0.46      |3.87      |0.60    |3.41    |0.001   |
|Melancholy      |3.83        |0.65      |3.83      |0.55    |-0.02   |0.987   |
|Optimism        |4.07        |0.66      |3.92      |0.60    |1.23    |0.220   |
|Seriousness     |4.06        |0.71      |4.32      |0.64    |-1.96   |0.053   |
|Trustworthiness |4.18        |0.41      |3.83      |0.53    |3.68    |0.000   |
|Vicious         |3.71        |0.69      |3.75      |0.69    |-0.28   |0.783   |
```

# Principal Components Analyses


```
# prep data format for PCA
rating_average_wide_clean <- data_rating %>% 
  group_by(face_sex,trait_rating,face_id) %>%
  summarise(rating.m=mean(rating.response)) %>%
  filter(trait_rating != "Commonness" & trait_rating != "Dullness") %>%
  spread(trait_rating, rating.m)
```


## PCA male faces, including dominance


```
pca.male<- rating_average_wide_clean %>% filter(face_sex=="male")
ev <- eigen(cor(pca.male[,-c(1:2)]))
nfactors <- length(ev$values[ev$values > 1])
fit.male <- principal(
  pca.male[,-c(1:2)],
  nfactors = nfactors,
  rotate="none", 
  scores=TRUE
)
fit.male.scores <- cbind(pca.male[,2],fit.male$scores)
kable(fit.male$loadings %>% unclass() %>% data.frame(), digits=3)
```


```
|                |    PC1|    PC2|
|:---------------|------:|------:|
|Attractiveness  |  0.612|  0.701|
|Cheerfulness    |  0.886|  0.112|
|Dominance       | -0.761|  0.336|
|Easygoingness   |  0.939| -0.089|
|Friendliness    |  0.956| -0.035|
|Ingenuousness   |  0.876| -0.167|
|Intelligence    |  0.380|  0.824|
|Kindness        |  0.904| -0.017|
|Melancholy      | -0.817| -0.042|
|Optimism        |  0.919|  0.122|
|Seriousness     | -0.773|  0.376|
|Trustworthiness |  0.773|  0.082|
|Vicious         | -0.869|  0.259|
```

## PCA female faces, including dominance


```
pca.female<- rating_average_wide_clean %>% filter(face_sex=="female")
ev <- eigen(cor(pca.female[,-c(1:2)] ))
nfactors <- length(ev$values[ev$values > 1])
fit.female <-  principal(
  pca.female[,-c(1:2)],
  nfactors = nfactors,
  rotate="none", 
  scores=TRUE
)
fit.female.scores <- cbind(pca.female[,2],fit.female$scores)
kable(fit.female$loadings %>% unclass() %>% data.frame(),digits=3)
```


```
|                |    PC1|    PC2|
|:---------------|------:|------:|
|Attractiveness  |  0.425|  0.826|
|Cheerfulness    |  0.924| -0.059|
|Dominance       | -0.903|  0.163|
|Easygoingness   |  0.955|  0.042|
|Friendliness    |  0.946| -0.092|
|Ingenuousness   |  0.818| -0.159|
|Intelligence    |  0.420|  0.839|
|Kindness        |  0.921| -0.039|
|Melancholy      | -0.884|  0.005|
|Optimism        |  0.925| -0.017|
|Seriousness     | -0.901|  0.160|
|Trustworthiness |  0.875| -0.013|
|Vicious         | -0.925|  0.121|
```

## PCA male faces, not including dominance


```
pca.male.no.dom <- pca.male[,-5]
ev <- eigen(cor(pca.male.no.dom[,-c(1:2)]))
nfactors <- length(ev$values[ev$values > 1])
fit.male.no.dom <- principal(
  pca.male.no.dom[,-c(1:2)],
  nfactors = nfactors,
  rotate="none", 
  scores=TRUE
)
fit.male.nodom.scores <- cbind(pca.male[,2],fit.male.no.dom$scores)
kable(fit.male.no.dom$loadings %>% unclass()%>% data.frame(),digits=3)
```


```
|                |    PC1|    PC2|
|:---------------|------:|------:|
|Attractiveness  |  0.631|  0.689|
|Cheerfulness    |  0.894|  0.068|
|Easygoingness   |  0.930| -0.100|
|Friendliness    |  0.959| -0.080|
|Ingenuousness   |  0.874| -0.212|
|Intelligence    |  0.397|  0.847|
|Kindness        |  0.907| -0.062|
|Melancholy      | -0.826|  0.010|
|Optimism        |  0.929|  0.072|
|Seriousness     | -0.756|  0.380|
|Trustworthiness |  0.783|  0.027|
|Vicious         | -0.850|  0.250|
```

## PCA female faces, not including dominance


```
pca.female.no.dom <- pca.female[,-5]
ev <- eigen(cor(pca.female.no.dom[,-c(1:2)] ))
nfactors <- length(ev$values[ev$values > 1])
fit.female.no.dom <-  principal(
  pca.female.no.dom[,-c(1:2)],
  nfactors = nfactors,
  rotate="none", 
  scores=TRUE
)
fit.female.nodom.scores <- cbind(pca.female[,2],fit.female.no.dom$scores)
kable(fit.female.no.dom$loadings %>% unclass() %>% data.frame(),digits=3)
```


```
|                |    PC1|    PC2|
|:---------------|------:|------:|
|Attractiveness  |  0.438|  0.824|
|Cheerfulness    |  0.929| -0.087|
|Easygoingness   |  0.957|  0.023|
|Friendliness    |  0.940| -0.104|
|Ingenuousness   |  0.813| -0.168|
|Intelligence    |  0.436|  0.828|
|Kindness        |  0.920| -0.054|
|Melancholy      | -0.893|  0.037|
|Optimism        |  0.928| -0.043|
|Seriousness     | -0.903|  0.186|
|Trustworthiness |  0.870| -0.020|
|Vicious         | -0.919|  0.131|
```

# Additional analyses for face ratings from male and female participants


```
# prep data format for PCA
rating_average_wide_clean_partsex <- data_rating %>% 
  group_by(face_sex,trait_rating,face_id, part_sex) %>%
  summarise(rating.m=mean(rating.response)) %>%
  filter(trait_rating != "Commonness" & trait_rating != "Dullness") %>%
  spread(trait_rating, rating.m)
```


## PCA male faces by male raters, including dominance


```
pca.male.male <- rating_average_wide_clean_partsex %>% 
  filter(face_sex == "male") %>%
  filter(part_sex == "male")
ev <- eigen(cor(pca.male.male[,-c(1:3)]))
nfactors <- length(ev$values[ev$values > 1])
fit.male.male <- principal(
  pca.male.male[,-c(1:3)],
  nfactors = nfactors,
  rotate="none", 
  scores=TRUE)
fit.male.scores <- cbind(pca.male.male[,3],fit.male.male$scores)
kable(fit.male.male$loadings %>% unclass() %>%data.frame(), digits=3)
```


```
|                |    PC1|    PC2|
|:---------------|------:|------:|
|Attractiveness  |  0.609|  0.646|
|Cheerfulness    |  0.776|  0.083|
|Dominance       | -0.593|  0.370|
|Easygoingness   |  0.837| -0.059|
|Friendliness    |  0.866|  0.042|
|Ingenuousness   |  0.768| -0.357|
|Intelligence    |  0.384|  0.744|
|Kindness        |  0.885| -0.007|
|Melancholy      | -0.602| -0.166|
|Optimism        |  0.835|  0.248|
|Seriousness     | -0.700|  0.430|
|Trustworthiness |  0.704| -0.037|
|Vicious         | -0.767|  0.273|
```

## PCA male faces by female raters, including dominance


```
pca.male.female <- rating_average_wide_clean_partsex %>% 
  filter(face_sex == "male") %>%
  filter(part_sex == "female")
ev <- eigen(cor(pca.male.female[,-c(1:3)]))
nfactors <- length(ev$values[ev$values > 1])
fit.male.female <- principal(
  pca.male.female[,-c(1:3)],
  nfactors = nfactors,
  rotate="none", 
  scores=TRUE)
fit.male.scores <- cbind(pca.male.female[,3],fit.male.female$scores)
kable(fit.male.female$loadings %>% unclass() %>%data.frame(), digits=3)
```


```
|                |    PC1|    PC2|
|:---------------|------:|------:|
|Attractiveness  |  0.590|  0.735|
|Cheerfulness    |  0.891|  0.060|
|Dominance       | -0.839|  0.265|
|Easygoingness   |  0.920| -0.117|
|Friendliness    |  0.938| -0.083|
|Ingenuousness   |  0.856|  0.022|
|Intelligence    |  0.342|  0.800|
|Kindness        |  0.842| -0.034|
|Melancholy      | -0.864|  0.022|
|Optimism        |  0.914| -0.059|
|Seriousness     | -0.737|  0.327|
|Trustworthiness |  0.775|  0.250|
|Vicious         | -0.883|  0.252|
```

## PCA female faces by male raters, including dominance


```
pca.female.male <- rating_average_wide_clean_partsex %>% 
  filter(face_sex == "female") %>%
  filter(part_sex == "male")
ev <- eigen(cor(pca.female.male[,-c(1:3)]))
nfactors <- length(ev$values[ev$values > 1])
fit.female.male <- principal(
  pca.female.male[,-c(1:3)],
  nfactors = nfactors,
  rotate="none", 
  scores=TRUE)
fit.female.scores <- cbind(pca.female.male[,3],fit.female.male$scores)
kable(fit.female.male$loadings %>% unclass() %>%data.frame(), digits=3)
```


```
|                |    PC1|    PC2|
|:---------------|------:|------:|
|Attractiveness  |  0.443|  0.793|
|Cheerfulness    |  0.868| -0.192|
|Dominance       | -0.784|  0.252|
|Easygoingness   |  0.920|  0.081|
|Friendliness    |  0.850| -0.171|
|Ingenuousness   |  0.661| -0.158|
|Intelligence    |  0.529|  0.620|
|Kindness        |  0.787| -0.040|
|Melancholy      | -0.852| -0.058|
|Optimism        |  0.877| -0.104|
|Seriousness     | -0.874|  0.185|
|Trustworthiness |  0.578|  0.459|
|Vicious         | -0.847|  0.200|
```

## PCA female faces by female raters, including dominance


```
pca.female.female <- rating_average_wide_clean_partsex %>% 
  filter(face_sex == "female") %>%
  filter(part_sex == "female")
ev <- eigen(cor(pca.female.female[,-c(1:3)]))
nfactors <- length(ev$values[ev$values > 1])
fit.female.female <- principal(
  pca.female.female[,-c(1:3)],
  nfactors = nfactors,
  rotate="none", 
  scores=TRUE)
fit.female.scores <- cbind(pca.female.female[,3],fit.female.female$scores)
kable(fit.female.female$loadings %>% unclass() %>%data.frame(), digits=3)
```


```
|                |    PC1|    PC2|
|:---------------|------:|------:|
|Attractiveness  |  0.407|  0.798|
|Cheerfulness    |  0.924|  0.057|
|Dominance       | -0.910|  0.162|
|Easygoingness   |  0.927| -0.006|
|Friendliness    |  0.937|  0.026|
|Ingenuousness   |  0.849| -0.116|
|Intelligence    |  0.192|  0.934|
|Kindness        |  0.911|  0.011|
|Melancholy      | -0.834|  0.004|
|Optimism        |  0.936|  0.049|
|Seriousness     | -0.844|  0.099|
|Trustworthiness |  0.873| -0.231|
|Vicious         | -0.905|  0.106|
```

## PCA male faces by male raters, not including dominance


```
pca.male.male.no.dom <- pca.male.male %>%
  select(-Dominance)
ev <- eigen(cor(pca.male.male.no.dom[,-c(1:3)]))
nfactors <- length(ev$values[ev$values > 1])
fit.male.male.no.dom <- principal(
  pca.male.male.no.dom[,-c(1:3)],
  nfactors = nfactors,
  rotate="none", 
  scores=TRUE)
fit.male.male.nodom.scores <- cbind(pca.male.male.no.dom[,2],fit.male.male.no.dom$scores)
kable(fit.male.male.no.dom$loadings %>% unclass()%>% data.frame(),digits=3)
```


```
|                |    PC1|    PC2|
|:---------------|------:|------:|
|Attractiveness  |  0.626|  0.630|
|Cheerfulness    |  0.790|  0.020|
|Easygoingness   |  0.826| -0.047|
|Friendliness    |  0.876| -0.025|
|Ingenuousness   |  0.763| -0.419|
|Intelligence    |  0.391|  0.806|
|Kindness        |  0.883| -0.034|
|Melancholy      | -0.613| -0.115|
|Optimism        |  0.849|  0.194|
|Seriousness     | -0.681|  0.414|
|Trustworthiness |  0.720| -0.132|
|Vicious         | -0.745|  0.231|
```

## PCA male faces by female raters, not including dominance


```
pca.male.female.no.dom <- pca.male.female %>%
  select(-Dominance)
ev <- eigen(cor(pca.male.female.no.dom[,-c(1:3)]))
nfactors <- length(ev$values[ev$values > 1])
fit.male.female.no.dom <- principal(
  pca.male.female.no.dom[,-c(1:3)],
  nfactors = nfactors,
  rotate="none", 
  scores=TRUE)
fit.male.female.nodom.scores <- cbind(pca.male.female.no.dom[,2],fit.male.female.no.dom$scores)
kable(fit.male.female.no.dom$loadings %>% unclass()%>% data.frame(),digits=3)
```


```
|                |    PC1|    PC2|
|:---------------|------:|------:|
|Attractiveness  |  0.611|  0.719|
|Cheerfulness    |  0.897|  0.017|
|Easygoingness   |  0.912| -0.134|
|Friendliness    |  0.939| -0.118|
|Ingenuousness   |  0.857| -0.002|
|Intelligence    |  0.362|  0.797|
|Kindness        |  0.842| -0.059|
|Melancholy      | -0.871|  0.071|
|Optimism        |  0.919| -0.104|
|Seriousness     | -0.730|  0.360|
|Trustworthiness |  0.783|  0.229|
|Vicious         | -0.864|  0.252|
```

## PCA female faces by male raters, not including dominance


```
pca.female.male.no.dom <- pca.female.male %>%
  select(-Dominance)
ev <- eigen(cor(pca.female.male.no.dom[,-c(1:3)]))
nfactors <- length(ev$values[ev$values > 1])
fit.female.male.no.dom <- principal(
  pca.female.male.no.dom[,-c(1:3)],
  nfactors = nfactors,
  rotate="none", 
  scores=TRUE)
fit.female.male.nodom.scores <- cbind(pca.female.male.no.dom[,2],fit.female.male.no.dom$scores)
kable(fit.female.male.no.dom$loadings %>% unclass()%>% data.frame(),digits=3)
```


```
|                |    PC1|    PC2|
|:---------------|------:|------:|
|Attractiveness  |  0.462|  0.788|
|Cheerfulness    |  0.874| -0.241|
|Easygoingness   |  0.922|  0.058|
|Friendliness    |  0.836| -0.173|
|Ingenuousness   |  0.653| -0.166|
|Intelligence    |  0.543|  0.611|
|Kindness        |  0.794| -0.081|
|Melancholy      | -0.862| -0.011|
|Optimism        |  0.881| -0.148|
|Seriousness     | -0.870|  0.213|
|Trustworthiness |  0.591|  0.439|
|Vicious         | -0.836|  0.208|
```

## PCA female faces by female raters, not including dominance


```
pca.female.female.no.dom <- pca.female.female %>%
  select(-Dominance)
ev <- eigen(cor(pca.female.female.no.dom[,-c(1:3)]))
nfactors <- length(ev$values[ev$values > 1])
fit.female.female.no.dom <- principal(
  pca.female.female.no.dom[,-c(1:3)],
  nfactors = nfactors,
  rotate="none", 
  scores=TRUE)
fit.female.female.nodom.scores <- cbind(pca.female.female.no.dom[,2],fit.female.female.no.dom$scores)
kable(fit.female.female.no.dom$loadings %>% unclass()%>% data.frame(),digits=3)
```


```
|                |    PC1|    PC2|
|:---------------|------:|------:|
|Attractiveness  |  0.417|  0.802|
|Cheerfulness    |  0.929|  0.030|
|Easygoingness   |  0.929| -0.028|
|Friendliness    |  0.936|  0.008|
|Ingenuousness   |  0.844| -0.126|
|Intelligence    |  0.215|  0.923|
|Kindness        |  0.908|  0.000|
|Melancholy      | -0.843|  0.038|
|Optimism        |  0.939|  0.024|
|Seriousness     | -0.851|  0.132|
|Trustworthiness |  0.863| -0.240|
|Vicious         | -0.899|  0.118|
```

LS0tCnRpdGxlOiAiQWRkaXRpb25hbCBhbmFseXNlcyBmb3IgZmFjZSByYXRpbmdzIGZyb20gbWFsZSBhbmQgZmVtYWxlIHBhcnRpY2lwYW50cyIKYXV0aG9yOiAiSG9uZ3lpIFdhbmcgZXQgYWwuIgpvdXRwdXQ6CiAgaHRtbF9ub3RlYm9vazoKICAgIHRvYzogdHJ1ZQotLS0KCgoKYGBge3IgbG9hZF9wYWNrYWdlcywgbWVzc2FnZT1GQUxTRX0KbGlicmFyeShwc3ljaCkKbGlicmFyeShicm9tYW4pICMgZm9yIHNlbnNpYmxlIHJvdW5kaW5nIGZ1bmN0aW9uCmxpYnJhcnkoa25pdHIpCmxpYnJhcnkodGlkeXZlcnNlKSAKYGBgCgpgYGB7ciwgbWVzc2FnZT1GQUxTRX0KZGF0YV9yYXRpbmcgPC0gcmVhZF9jc3YoImNoaW5lc2VfcmF0aW5nX3Jhdy5jc3YiKQpgYGAKCgojIyMgUmF0aW5nIHRhc2sgZGVtb2dyYXBoaWNzCmBgYHtyfQojIyMgZ2V0IGFnZSBhbmQgc2V4IGluZm8gCgpkYXRhX3JhdGluZyAlPiUgCiAgZ3JvdXBfYnkocGFydGljaXBhbnRfaWQsIHBhcnRfc2V4KSAlPiUKICBzdW1tYXJpc2UoYWdlPW1lYW4ocGFydF9hZ2UpKSAlPiUKICB1bmdyb3VwKCkgJT4lCiAgZ3JvdXBfYnkocGFydF9zZXgpJT4lCiAgc3VtbWFyaXplKGFnZS5tPW15cm91bmQobWVhbihhZ2UpLDIpLAogICAgICAgICAgICBhZ2Uuc2Q9bXlyb3VuZChzZChhZ2UpLDIpLAogICAgICAgICAgICBjb3VudD1uKCkpICU+JQogIHVuZ3JvdXAoKSAlPiUKICBrYWJsZSgpCgpgYGAKCiMjIENyb25iYWNoJ3MgYWxwaGEgb2YgcmF0aW5ncwpgYGB7ciBhbHBoYXMsIGVjaG89RkFMU0UsIHdhcm5pbmcgPSBGQUxTRX0KIAojIyBDcm9uYmFjaCdzIGFscGhhIG9mIHJhdGluZ3MKYm9vdC5pdGVyID0gMTAwMAoKIyMgY2FsY3VsYXRlIGFscGhhcyBmb3IgZWFjaCBqdWRnZW1lbnQvZmFjZV9zZXggY29tYm8KZ2V0QWxwaGFzIDwtIGZ1bmN0aW9uKHVzZXJfaWQsIHN0aW11bHVzLCByYXRpbmcpIHsKICBkYXRhIDwtIGRhdGEuZnJhbWUodXNlcl9pZCA9IHVzZXJfaWQsCiAgICAgICAgICAgICAgICAgICAgIHN0aW11bHVzID0gc3RpbXVsdXMsCiAgICAgICAgICAgICAgICAgICAgIHJhdGluZyA9IHJhdGluZykKICAKICBjYXB0dXJlLm91dHB1dChzdXBwcmVzc1dhcm5pbmdzKAogICAgYSA8LSBkYXRhICU+JQogICAgICBzcHJlYWQodXNlcl9pZCwgcmF0aW5nKSAlPiUKICAgICAgcHN5Y2g6OmFscGhhKGNoZWNrLmtleXMgPSBGQUxTRSwgbi5pdGVyID0gYm9vdC5pdGVyLCB3YXJuaW5ncyA9IEZBTFNFKQogICkpCiAgIyBib290cyA8LSBwYXN0ZTAobXlyb3VuZChhJHRvdGFsJHN0ZC5hbHBoYSwyKSwgCiAgIyAgICAgICAgICAgICAgICAgIiBbIiwgbXlyb3VuZChhJGJvb3QuY2lbMV0sMiksIAogICMgICAgICAgICAgICAgICAgICItIiwgbXlyb3VuZChhJGJvb3QuY2lbM10sMiksICJdIikKICBib290cyA8LSBteXJvdW5kKGEkdG90YWwkc3RkLmFscGhhLDIpCiAgcmV0dXJuKGJvb3RzKQp9CgpzZXQuc2VlZCgzKQoKYWxwaGFzIDwtIGRhdGFfcmF0aW5nICU+JQogIGdyb3VwX2J5KGZhY2Vfc2V4LHRyYWl0X3JhdGluZykgJT4lCiAgc3VtbWFyaXNlKGFscGhhPWdldEFscGhhcyhwYXJ0aWNpcGFudF9pZCwgZmFjZV9pZCwgcmF0aW5nLnJlc3BvbnNlKSkgJT4lCiAgc2VsZWN0KHRyYWl0X3JhdGluZywgZmFjZV9zZXgsIGFscGhhKQoKbWluLmZhY2UuYWxwaGEgPC0gYWxwaGFzICU+JQogIGdyb3VwX2J5KCkgJT4lCiAgc3VtbWFyaXNlKG1pbiA9IG1pbihhbHBoYSkpICU+JSAKICB1bmdyb3VwKCkgJT4lCiAgYXMubnVtZXJpYygpCgpsb3cuYWxwaGFzIDwtIGFscGhhcyAlPiUKICBmaWx0ZXIoYWxwaGEgPCAwLjcpCgpgYGAKCmBgYHtyIGFscGhhcy10YWJsZSwgZWNobz1GQUxTRSwgd2FybmluZz1GQUxTRSwgbWVzc2FnZT1GQUxTRX0KCiMgZm9ybWF0IGZvciB0YWJsZSAKYWxwaGFzLnRhYmxlIDwtIGFscGhhcyAlPiUKICB1bml0ZSh0ZW1wLCBmYWNlX3NleCkgJT4lCiAgc3ByZWFkKHRlbXAsIGFscGhhKSAlPiUKICByZW5hbWUoVHJhaXQgPSB0cmFpdF9yYXRpbmcsCiAgICAgICAgICJNYWxlIEZhY2UiID0gbWFsZSwKICAgICAgICAgIkZlbWFsZSBGYWNlIiA9IGZlbWFsZSkgJT4lCiAgc2VsZWN0KGBUcmFpdGAsIGBNYWxlIEZhY2VgLCBgRmVtYWxlIEZhY2VgKSNzd2l0Y2hlcyBjb2x1bW4gb3JkZXIgYXJvdW5kIAoKa2FibGUoYWxwaGFzLnRhYmxlLCBjYXB0aW9uPSIqVGFibGUgMS4qIEludGVyLXJhdGVyIHJlbGlhYmlsaXR5IChDcm9uYmFjaCdzIGFscGhhKSBmb3IgdHJhaXQgcmF0aW5ncyBvZiBmYWNlcy4iKQpgYGAKCiMjIERlc2NyaXB0aXZlIHN0YXRpc3RpY3MgZm9yIGFsbCB0cmFpdHMgCmBgYHtyLCBlY2hvPUZBTFNFfQpkYXRhX3JhdGluZyAlPiUKICBncm91cF9ieShmYWNlX3NleCx0cmFpdF9yYXRpbmcsZmFjZV9pZCkgJT4lCiAgc3VtbWFyaXNlKHJhdGluZy5tPW1lYW4ocmF0aW5nLnJlc3BvbnNlKSkgJT4lCiAgZ3JvdXBfYnkodHJhaXRfcmF0aW5nKSAlPiUKICBuZXN0KCkgJT4lCiAgbXV0YXRlKHQgPSBwdXJycjo6bWFwKGRhdGEsIGZ1bmN0aW9uKGQpIHsKICAgIHR0ZXN0IDwtIGQgJT4lIHQudGVzdChyYXRpbmcubX5mYWNlX3NleCwgZGF0YSA9IC4pICU+JQogICAgICBicm9vbTo6dGlkeSgpICU+JQogICAgICBzZWxlY3QoInQudmFsdWUiID0gc3RhdGlzdGljLCBwLnZhbHVlKSAlPiUKICAgICAgbXV0YXRlKAogICAgICAgIHQudmFsdWUgPSBteXJvdW5kKHQudmFsdWUsIDIpLAogICAgICAgIHAudmFsdWUgPSBteXJvdW5kKHAudmFsdWUsIDMpCiAgICAgICkKICAgIAogICAgZCAlPiUKICAgICAgZ3JvdXBfYnkoZmFjZV9zZXgpICU+JQogICAgICBzdW1tYXJpc2UoCiAgICAgICAgbWVhbiA9IG1lYW4ocmF0aW5nLm0pLCAKICAgICAgICBzZCA9IHNkKHJhdGluZy5tKQogICAgICApICU+JQogICAgICBnYXRoZXIoInN0YXQiLCAidmFsIiwgbWVhbjpzZCkgJT4lCiAgICAgIHVuaXRlKCJ2YXIiLCBmYWNlX3NleCwgc3RhdCwgc2VwID0gIi4iKSAlPiUKICAgICAgc3ByZWFkKHZhciwgdmFsKSAlPiUKICAgICAgbXV0YXRlX2FsbChteXJvdW5kLCAyKSAlPiUKICAgICAgYmluZF9jb2xzKHR0ZXN0KQogIH0pKSAlPiUKICBzZWxlY3QoLWRhdGEpICU+JQogIHVubmVzdCh0KSAlPiUKICBrYWJsZSgpCmBgYAoKIyBQcmluY2lwYWwgQ29tcG9uZW50cyBBbmFseXNlcwpgYGB7cn0KIyBwcmVwIGRhdGEgZm9ybWF0IGZvciBQQ0EKcmF0aW5nX2F2ZXJhZ2Vfd2lkZV9jbGVhbiA8LSBkYXRhX3JhdGluZyAlPiUgCiAgZ3JvdXBfYnkoZmFjZV9zZXgsdHJhaXRfcmF0aW5nLGZhY2VfaWQpICU+JQogIHN1bW1hcmlzZShyYXRpbmcubT1tZWFuKHJhdGluZy5yZXNwb25zZSkpICU+JQogIGZpbHRlcih0cmFpdF9yYXRpbmcgIT0gIkNvbW1vbm5lc3MiICYgdHJhaXRfcmF0aW5nICE9ICJEdWxsbmVzcyIpICU+JQogIHNwcmVhZCh0cmFpdF9yYXRpbmcsIHJhdGluZy5tKQpgYGAKCgojIyBQQ0EgbWFsZSBmYWNlcywgaW5jbHVkaW5nIGRvbWluYW5jZQpgYGB7ciB9CnBjYS5tYWxlPC0gcmF0aW5nX2F2ZXJhZ2Vfd2lkZV9jbGVhbiAlPiUgZmlsdGVyKGZhY2Vfc2V4PT0ibWFsZSIpCgpldiA8LSBlaWdlbihjb3IocGNhLm1hbGVbLC1jKDE6MildKSkKbmZhY3RvcnMgPC0gbGVuZ3RoKGV2JHZhbHVlc1tldiR2YWx1ZXMgPiAxXSkKCmZpdC5tYWxlIDwtIHByaW5jaXBhbCgKICBwY2EubWFsZVssLWMoMToyKV0sCiAgbmZhY3RvcnMgPSBuZmFjdG9ycywKICByb3RhdGU9Im5vbmUiLCAKICBzY29yZXM9VFJVRQopCgpmaXQubWFsZS5zY29yZXMgPC0gY2JpbmQocGNhLm1hbGVbLDJdLGZpdC5tYWxlJHNjb3JlcykKa2FibGUoZml0Lm1hbGUkbG9hZGluZ3MgJT4lIHVuY2xhc3MoKSAlPiUgZGF0YS5mcmFtZSgpLCBkaWdpdHM9MykgCgpgYGAKCgojIyBQQ0EgZmVtYWxlIGZhY2VzLCBpbmNsdWRpbmcgZG9taW5hbmNlCmBgYHtyfQoKcGNhLmZlbWFsZTwtIHJhdGluZ19hdmVyYWdlX3dpZGVfY2xlYW4gJT4lIGZpbHRlcihmYWNlX3NleD09ImZlbWFsZSIpCgpldiA8LSBlaWdlbihjb3IocGNhLmZlbWFsZVssLWMoMToyKV0gKSkKbmZhY3RvcnMgPC0gbGVuZ3RoKGV2JHZhbHVlc1tldiR2YWx1ZXMgPiAxXSkKCmZpdC5mZW1hbGUgPC0gIHByaW5jaXBhbCgKICBwY2EuZmVtYWxlWywtYygxOjIpXSwKICBuZmFjdG9ycyA9IG5mYWN0b3JzLAogIHJvdGF0ZT0ibm9uZSIsIAogIHNjb3Jlcz1UUlVFCikKCmZpdC5mZW1hbGUuc2NvcmVzIDwtIGNiaW5kKHBjYS5mZW1hbGVbLDJdLGZpdC5mZW1hbGUkc2NvcmVzKQprYWJsZShmaXQuZmVtYWxlJGxvYWRpbmdzICU+JSB1bmNsYXNzKCkgJT4lIGRhdGEuZnJhbWUoKSxkaWdpdHM9MykKCmBgYAoKIyMgUENBIG1hbGUgZmFjZXMsIG5vdCBpbmNsdWRpbmcgZG9taW5hbmNlCmBgYHtyIH0KcGNhLm1hbGUubm8uZG9tIDwtIHBjYS5tYWxlWywtNV0KCmV2IDwtIGVpZ2VuKGNvcihwY2EubWFsZS5uby5kb21bLC1jKDE6MildKSkKbmZhY3RvcnMgPC0gbGVuZ3RoKGV2JHZhbHVlc1tldiR2YWx1ZXMgPiAxXSkKZml0Lm1hbGUubm8uZG9tIDwtIHByaW5jaXBhbCgKICBwY2EubWFsZS5uby5kb21bLC1jKDE6MildLAogIG5mYWN0b3JzID0gbmZhY3RvcnMsCiAgcm90YXRlPSJub25lIiwgCiAgc2NvcmVzPVRSVUUKKQoKZml0Lm1hbGUubm9kb20uc2NvcmVzIDwtIGNiaW5kKHBjYS5tYWxlWywyXSxmaXQubWFsZS5uby5kb20kc2NvcmVzKQoKa2FibGUoZml0Lm1hbGUubm8uZG9tJGxvYWRpbmdzICU+JSB1bmNsYXNzKCklPiUgZGF0YS5mcmFtZSgpLGRpZ2l0cz0zKQpgYGAKCiMjIFBDQSBmZW1hbGUgZmFjZXMsIG5vdCBpbmNsdWRpbmcgZG9taW5hbmNlCmBgYHtyfQoKcGNhLmZlbWFsZS5uby5kb20gPC0gcGNhLmZlbWFsZVssLTVdCmV2IDwtIGVpZ2VuKGNvcihwY2EuZmVtYWxlLm5vLmRvbVssLWMoMToyKV0gKSkKbmZhY3RvcnMgPC0gbGVuZ3RoKGV2JHZhbHVlc1tldiR2YWx1ZXMgPiAxXSkKCmZpdC5mZW1hbGUubm8uZG9tIDwtICBwcmluY2lwYWwoCiAgcGNhLmZlbWFsZS5uby5kb21bLC1jKDE6MildLAogIG5mYWN0b3JzID0gbmZhY3RvcnMsCiAgcm90YXRlPSJub25lIiwgCiAgc2NvcmVzPVRSVUUKKQoKZml0LmZlbWFsZS5ub2RvbS5zY29yZXMgPC0gY2JpbmQocGNhLmZlbWFsZVssMl0sZml0LmZlbWFsZS5uby5kb20kc2NvcmVzKQoKa2FibGUoZml0LmZlbWFsZS5uby5kb20kbG9hZGluZ3MgJT4lIHVuY2xhc3MoKSAlPiUgZGF0YS5mcmFtZSgpLGRpZ2l0cz0zKQoKYGBgCgojIEFkZGl0aW9uYWwgYW5hbHlzZXMgZm9yIGZhY2UgcmF0aW5ncyBmcm9tIG1hbGUgYW5kIGZlbWFsZSBwYXJ0aWNpcGFudHMKYGBge3J9CiMgcHJlcCBkYXRhIGZvcm1hdCBmb3IgUENBCnJhdGluZ19hdmVyYWdlX3dpZGVfY2xlYW5fcGFydHNleCA8LSBkYXRhX3JhdGluZyAlPiUgCiAgZ3JvdXBfYnkoZmFjZV9zZXgsdHJhaXRfcmF0aW5nLGZhY2VfaWQsIHBhcnRfc2V4KSAlPiUKICBzdW1tYXJpc2UocmF0aW5nLm09bWVhbihyYXRpbmcucmVzcG9uc2UpKSAlPiUKICBmaWx0ZXIodHJhaXRfcmF0aW5nICE9ICJDb21tb25uZXNzIiAmIHRyYWl0X3JhdGluZyAhPSAiRHVsbG5lc3MiKSAlPiUKICBzcHJlYWQodHJhaXRfcmF0aW5nLCByYXRpbmcubSkKYGBgCgoKIyMgUENBIG1hbGUgZmFjZXMgYnkgbWFsZSByYXRlcnMsIGluY2x1ZGluZyBkb21pbmFuY2UKYGBge3IgfQoKcGNhLm1hbGUubWFsZSA8LSByYXRpbmdfYXZlcmFnZV93aWRlX2NsZWFuX3BhcnRzZXggJT4lIAogIGZpbHRlcihmYWNlX3NleCA9PSAibWFsZSIpICU+JQogIGZpbHRlcihwYXJ0X3NleCA9PSAibWFsZSIpCgpldiA8LSBlaWdlbihjb3IocGNhLm1hbGUubWFsZVssLWMoMTozKV0pKQpuZmFjdG9ycyA8LSBsZW5ndGgoZXYkdmFsdWVzW2V2JHZhbHVlcyA+IDFdKQoKZml0Lm1hbGUubWFsZSA8LSBwcmluY2lwYWwoCiAgcGNhLm1hbGUubWFsZVssLWMoMTozKV0sCiAgbmZhY3RvcnMgPSBuZmFjdG9ycywKICByb3RhdGU9Im5vbmUiLCAKICBzY29yZXM9VFJVRSkKCmZpdC5tYWxlLnNjb3JlcyA8LSBjYmluZChwY2EubWFsZS5tYWxlWywyXSxmaXQubWFsZS5tYWxlJHNjb3JlcykKa2FibGUoZml0Lm1hbGUubWFsZSRsb2FkaW5ncyAlPiUgdW5jbGFzcygpICU+JWRhdGEuZnJhbWUoKSwgZGlnaXRzPTMpIAoKYGBgCgojIyBQQ0EgbWFsZSBmYWNlcyBieSBmZW1hbGUgcmF0ZXJzLCBpbmNsdWRpbmcgZG9taW5hbmNlCmBgYHtyIH0KICAgIApwY2EubWFsZS5mZW1hbGUgPC0gcmF0aW5nX2F2ZXJhZ2Vfd2lkZV9jbGVhbl9wYXJ0c2V4ICU+JSAKICBmaWx0ZXIoZmFjZV9zZXggPT0gIm1hbGUiKSAlPiUKICBmaWx0ZXIocGFydF9zZXggPT0gImZlbWFsZSIpCgpldiA8LSBlaWdlbihjb3IocGNhLm1hbGUuZmVtYWxlWywtYygxOjMpXSkpCm5mYWN0b3JzIDwtIGxlbmd0aChldiR2YWx1ZXNbZXYkdmFsdWVzID4gMV0pCgpmaXQubWFsZS5mZW1hbGUgPC0gcHJpbmNpcGFsKAogIHBjYS5tYWxlLmZlbWFsZVssLWMoMTozKV0sCiAgbmZhY3RvcnMgPSBuZmFjdG9ycywKICByb3RhdGU9Im5vbmUiLCAKICBzY29yZXM9VFJVRSkKCmZpdC5tYWxlLnNjb3JlcyA8LSBjYmluZChwY2EubWFsZS5mZW1hbGVbLDJdLGZpdC5tYWxlLmZlbWFsZSRzY29yZXMpCmthYmxlKGZpdC5tYWxlLmZlbWFsZSRsb2FkaW5ncyAlPiUgdW5jbGFzcygpICU+JWRhdGEuZnJhbWUoKSwgZGlnaXRzPTMpIAoKYGBgCgojIyBQQ0EgZmVtYWxlIGZhY2VzIGJ5IG1hbGUgcmF0ZXJzLCBpbmNsdWRpbmcgZG9taW5hbmNlCmBgYHtyIH0KcGNhLmZlbWFsZS5tYWxlIDwtIHJhdGluZ19hdmVyYWdlX3dpZGVfY2xlYW5fcGFydHNleCAlPiUgCiAgZmlsdGVyKGZhY2Vfc2V4ID09ICJmZW1hbGUiKSAlPiUKICBmaWx0ZXIocGFydF9zZXggPT0gIm1hbGUiKQoKZXYgPC0gZWlnZW4oY29yKHBjYS5mZW1hbGUubWFsZVssLWMoMTozKV0pKQpuZmFjdG9ycyA8LSBsZW5ndGgoZXYkdmFsdWVzW2V2JHZhbHVlcyA+IDFdKQoKZml0LmZlbWFsZS5tYWxlIDwtIHByaW5jaXBhbCgKICBwY2EuZmVtYWxlLm1hbGVbLC1jKDE6MyldLAogIG5mYWN0b3JzID0gbmZhY3RvcnMsCiAgcm90YXRlPSJub25lIiwgCiAgc2NvcmVzPVRSVUUpCgpmaXQuZmVtYWxlLnNjb3JlcyA8LSBjYmluZChwY2EuZmVtYWxlLm1hbGVbLDJdLGZpdC5mZW1hbGUubWFsZSRzY29yZXMpCmthYmxlKGZpdC5mZW1hbGUubWFsZSRsb2FkaW5ncyAlPiUgdW5jbGFzcygpICU+JWRhdGEuZnJhbWUoKSwgZGlnaXRzPTMpIAoKYGBgCgojIyBQQ0EgZmVtYWxlIGZhY2VzIGJ5IGZlbWFsZSByYXRlcnMsIGluY2x1ZGluZyBkb21pbmFuY2UKYGBge3IgfQpwY2EuZmVtYWxlLmZlbWFsZSA8LSByYXRpbmdfYXZlcmFnZV93aWRlX2NsZWFuX3BhcnRzZXggJT4lIAogIGZpbHRlcihmYWNlX3NleCA9PSAiZmVtYWxlIikgJT4lCiAgZmlsdGVyKHBhcnRfc2V4ID09ICJmZW1hbGUiKQoKZXYgPC0gZWlnZW4oY29yKHBjYS5mZW1hbGUuZmVtYWxlWywtYygxOjMpXSkpCm5mYWN0b3JzIDwtIGxlbmd0aChldiR2YWx1ZXNbZXYkdmFsdWVzID4gMV0pCgpmaXQuZmVtYWxlLmZlbWFsZSA8LSBwcmluY2lwYWwoCiAgcGNhLmZlbWFsZS5mZW1hbGVbLC1jKDE6MyldLAogIG5mYWN0b3JzID0gbmZhY3RvcnMsCiAgcm90YXRlPSJub25lIiwgCiAgc2NvcmVzPVRSVUUpCgpmaXQuZmVtYWxlLnNjb3JlcyA8LSBjYmluZChwY2EuZmVtYWxlLmZlbWFsZVssMl0sZml0LmZlbWFsZS5mZW1hbGUkc2NvcmVzKQprYWJsZShmaXQuZmVtYWxlLmZlbWFsZSRsb2FkaW5ncyAlPiUgdW5jbGFzcygpICU+JWRhdGEuZnJhbWUoKSwgZGlnaXRzPTMpIAoKYGBgCgojIyBQQ0EgbWFsZSBmYWNlcyBieSBtYWxlIHJhdGVycywgbm90IGluY2x1ZGluZyBkb21pbmFuY2UKYGBge3IgfQpwY2EubWFsZS5tYWxlLm5vLmRvbSA8LSBwY2EubWFsZS5tYWxlICU+JQogIHNlbGVjdCgtRG9taW5hbmNlKQoKZXYgPC0gZWlnZW4oY29yKHBjYS5tYWxlLm1hbGUubm8uZG9tWywtYygxOjMpXSkpCm5mYWN0b3JzIDwtIGxlbmd0aChldiR2YWx1ZXNbZXYkdmFsdWVzID4gMV0pCgpmaXQubWFsZS5tYWxlLm5vLmRvbSA8LSBwcmluY2lwYWwoCiAgcGNhLm1hbGUubWFsZS5uby5kb21bLC1jKDE6MyldLAogIG5mYWN0b3JzID0gbmZhY3RvcnMsCiAgcm90YXRlPSJub25lIiwgCiAgc2NvcmVzPVRSVUUpCgpmaXQubWFsZS5tYWxlLm5vZG9tLnNjb3JlcyA8LSBjYmluZChwY2EubWFsZS5tYWxlLm5vLmRvbVssMl0sZml0Lm1hbGUubWFsZS5uby5kb20kc2NvcmVzKQprYWJsZShmaXQubWFsZS5tYWxlLm5vLmRvbSRsb2FkaW5ncyAlPiUgdW5jbGFzcygpJT4lIGRhdGEuZnJhbWUoKSxkaWdpdHM9MykKYGBgCgojIyBQQ0EgbWFsZSBmYWNlcyBieSBmZW1hbGUgcmF0ZXJzLCBub3QgaW5jbHVkaW5nIGRvbWluYW5jZQpgYGB7ciB9CnBjYS5tYWxlLmZlbWFsZS5uby5kb20gPC0gcGNhLm1hbGUuZmVtYWxlICU+JQogIHNlbGVjdCgtRG9taW5hbmNlKQoKZXYgPC0gZWlnZW4oY29yKHBjYS5tYWxlLmZlbWFsZS5uby5kb21bLC1jKDE6MyldKSkKbmZhY3RvcnMgPC0gbGVuZ3RoKGV2JHZhbHVlc1tldiR2YWx1ZXMgPiAxXSkKCmZpdC5tYWxlLmZlbWFsZS5uby5kb20gPC0gcHJpbmNpcGFsKAogIHBjYS5tYWxlLmZlbWFsZS5uby5kb21bLC1jKDE6MyldLAogIG5mYWN0b3JzID0gbmZhY3RvcnMsCiAgcm90YXRlPSJub25lIiwgCiAgc2NvcmVzPVRSVUUpCgpmaXQubWFsZS5mZW1hbGUubm9kb20uc2NvcmVzIDwtIGNiaW5kKHBjYS5tYWxlLmZlbWFsZS5uby5kb21bLDJdLGZpdC5tYWxlLmZlbWFsZS5uby5kb20kc2NvcmVzKQprYWJsZShmaXQubWFsZS5mZW1hbGUubm8uZG9tJGxvYWRpbmdzICU+JSB1bmNsYXNzKCklPiUgZGF0YS5mcmFtZSgpLGRpZ2l0cz0zKQpgYGAKCiMjIFBDQSBmZW1hbGUgZmFjZXMgYnkgbWFsZSByYXRlcnMsIG5vdCBpbmNsdWRpbmcgZG9taW5hbmNlCmBgYHtyIH0KcGNhLmZlbWFsZS5tYWxlLm5vLmRvbSA8LSBwY2EuZmVtYWxlLm1hbGUgJT4lCiAgc2VsZWN0KC1Eb21pbmFuY2UpCgpldiA8LSBlaWdlbihjb3IocGNhLmZlbWFsZS5tYWxlLm5vLmRvbVssLWMoMTozKV0pKQpuZmFjdG9ycyA8LSBsZW5ndGgoZXYkdmFsdWVzW2V2JHZhbHVlcyA+IDFdKQoKZml0LmZlbWFsZS5tYWxlLm5vLmRvbSA8LSBwcmluY2lwYWwoCiAgcGNhLmZlbWFsZS5tYWxlLm5vLmRvbVssLWMoMTozKV0sCiAgbmZhY3RvcnMgPSBuZmFjdG9ycywKICByb3RhdGU9Im5vbmUiLCAKICBzY29yZXM9VFJVRSkKCmZpdC5mZW1hbGUubWFsZS5ub2RvbS5zY29yZXMgPC0gY2JpbmQocGNhLmZlbWFsZS5tYWxlLm5vLmRvbVssMl0sZml0LmZlbWFsZS5tYWxlLm5vLmRvbSRzY29yZXMpCmthYmxlKGZpdC5mZW1hbGUubWFsZS5uby5kb20kbG9hZGluZ3MgJT4lIHVuY2xhc3MoKSU+JSBkYXRhLmZyYW1lKCksZGlnaXRzPTMpCgpgYGAKCiMjIFBDQSBmZW1hbGUgZmFjZXMgYnkgZmVtYWxlIHJhdGVycywgbm90IGluY2x1ZGluZyBkb21pbmFuY2UKYGBge3IgfQpwY2EuZmVtYWxlLmZlbWFsZS5uby5kb20gPC0gcGNhLmZlbWFsZS5mZW1hbGUgJT4lCiAgc2VsZWN0KC1Eb21pbmFuY2UpCgpldiA8LSBlaWdlbihjb3IocGNhLmZlbWFsZS5mZW1hbGUubm8uZG9tWywtYygxOjMpXSkpCm5mYWN0b3JzIDwtIGxlbmd0aChldiR2YWx1ZXNbZXYkdmFsdWVzID4gMV0pCgpmaXQuZmVtYWxlLmZlbWFsZS5uby5kb20gPC0gcHJpbmNpcGFsKAogIHBjYS5mZW1hbGUuZmVtYWxlLm5vLmRvbVssLWMoMTozKV0sCiAgbmZhY3RvcnMgPSBuZmFjdG9ycywKICByb3RhdGU9Im5vbmUiLCAKICBzY29yZXM9VFJVRSkKCmZpdC5mZW1hbGUuZmVtYWxlLm5vZG9tLnNjb3JlcyA8LSBjYmluZChwY2EuZmVtYWxlLmZlbWFsZS5uby5kb21bLDJdLGZpdC5mZW1hbGUuZmVtYWxlLm5vLmRvbSRzY29yZXMpCmthYmxlKGZpdC5mZW1hbGUuZmVtYWxlLm5vLmRvbSRsb2FkaW5ncyAlPiUgdW5jbGFzcygpJT4lIGRhdGEuZnJhbWUoKSxkaWdpdHM9MykKCmBgYA==
